# Obstructive sleep apnea, the NLRP3 inflammasome and the potential effects of incretin therapies

**DOI:** 10.3389/frsle.2024.1524593

**Published:** 2025-01-08

**Authors:** Michelle Wei, Jennifer A. Teske, Saif Mashaqi, Daniel Combs

**Affiliations:** ^1^University of Arizona Health Sciences Center for Sleep and Circadian Sciences, University of Arizona, Tucson, AZ, United States; ^2^School of Nutritional Sciences and Wellness, University of Arizona, Tucson, AZ, United States; ^3^Department of Medicine, University of Arizona, Tucson, AZ, United States; ^4^Department of Pediatrics, University of Arizona, Tucson, AZ, United States

**Keywords:** obstructive sleep apnea, inflammasome, intermittent hypoxemia, glucagon like peptide-1 receptor agonists, continuous positive airway pressure therapy

## Abstract

Obstructive sleep apnea (OSA) is a common sleep disorder associated with serious neurological and cardiovascular complications. Intermittent hypoxia and reoxygenation, a key feature of OSA, produces molecular signals that activate various inflammatory pathways, notably the inflammasome—a multiprotein complex that promotes the release of pro-inflammatory cytokines including IL-18 and IL-1β. This results in systemic inflammation, which contributes to the development of the neurological and cardiovascular complications seen in OSA. In this review, we will first examine the pathways through which intermittent hypoxia induces inflammasome activation. Then, we will connect the inflammasome to the downstream neurological and cardiovascular effects of OSA. Finally, we will explore potential interactions between the inflammasome and OSA treatments including Continuous Positive Airway Pressure therapy and glucagon like peptide-1 receptor agonists (GLP-1RAs).

## 1 Introduction

Obstructive sleep apnea (OSA) is characterized by repeated pharyngeal collapse during sleep, resulting in periods of intermittent hypoxia (IH), hypercapnia, and sleep fragmentation (Levy et al., 2015). IH contributes to the development of systemic complications, including neurocognitive impairment (Yang et al., 2013), pulmonary hypertension (Kholdani et al., 2015), and cardiovascular disease (Zdravkovic et al., 2022). OSA affects around 13% of men and 6% of women between the ages of 30–70 (Young et al., 2009). The prevalence of OSA is even higher in certain populations, including individuals with overweight or obesity where excess fatty tissues in the upper airway increases intrathoracic pressure, resulting in OSA (Jehan et al., 2017). Excess body weight is responsible for approximately 60% of moderate to severe cases in the U.S. (Patel, 2024). Obesity also contributes to the development of obesity hypoventilation syndrome (OHS), which is characterized by daytime hypoventilation and sleep-disordered breathing (SDB). It is estimated that OSA accounts for 90% of SDB in patients with OHS (Shetty and Parthasarathy, 2015). The interplay of excess adipose tissue, daytime hypoventilation, and OSA creates a pro-inflammatory state driven by intermittent hypoxia, oxidative stress, and the release of inflammatory mediators (Shetty and Parthasarathy, 2015; Borel et al., 2009; Ellulu et al., 2017).

As OSA is a significant public health concern, efforts have been made to understand its molecular pathology, with the goal of improving diagnosis and treatment. Recent studies suggest that the NLRP3 inflammasome is involved in the development of OSA comorbidities. Expressed predominantly in monocytes and macrophages, NLRP3 [nucleotide-binding oligomerization domain (NOD)-, leucine-rich repeat (LRR), and pyrin domain-containing protein 3] is an intracellular pattern-recognition receptor that is activated by damage-associated molecular patterns (DAMPs) (Swanson et al., 2019; Honda et al., 2023). Activation of NLRP3 results in the formation of the inflammasome, a protein complex consisting of NLRP3, ASC (apoptosis-associated speck-like protein containing a caspase recruitment domain), and caspase 1. The inflammasome induces an inflammatory response by cleaving immature forms of the cytokines IL-18 and IL-1β and promoting their release into circulation (Swanson et al., 2019).

OSA can generate DAMPs that activate the inflammasome (Gong et al., 2020; Diaz-Garcia et al., 2022), resulting in systemic inflammation associated with various neurological and cardiovascular comorbidities. In the central nervous system, OSA-induced intermittent hypoxia activates the NLRP3 inflammasome in microglia, the brain's resident immune cells. This activation leads to the release of pro-inflammatory cytokines, such as IL-1β and IL-18, which drive neuroinflammation and neuronal apoptosis (Gong et al., 2020; Wu et al., 2021; Zhang et al., 2023). These processes are associated with structural and functional changes in the hippocampus, resulting in cognition and memory deficits (Liu et al., 2020). In the periphery, inflammasome activation in monocytes and macrophages contributes to the release of inflammatory cytokines that promote endothelial injury (Kohler and Stradling, 2010; Gaisl et al., 2015), atherosclerosis (Diaz-Garcia et al., 2022, 2023), and pulmonary hypertension (Song et al., 2020; Fu et al., 2020).

## 2 Inflammasome activation in OSA

### 2.1 Inflammasome activation in monocytes and macrophages

**Figure 1:**
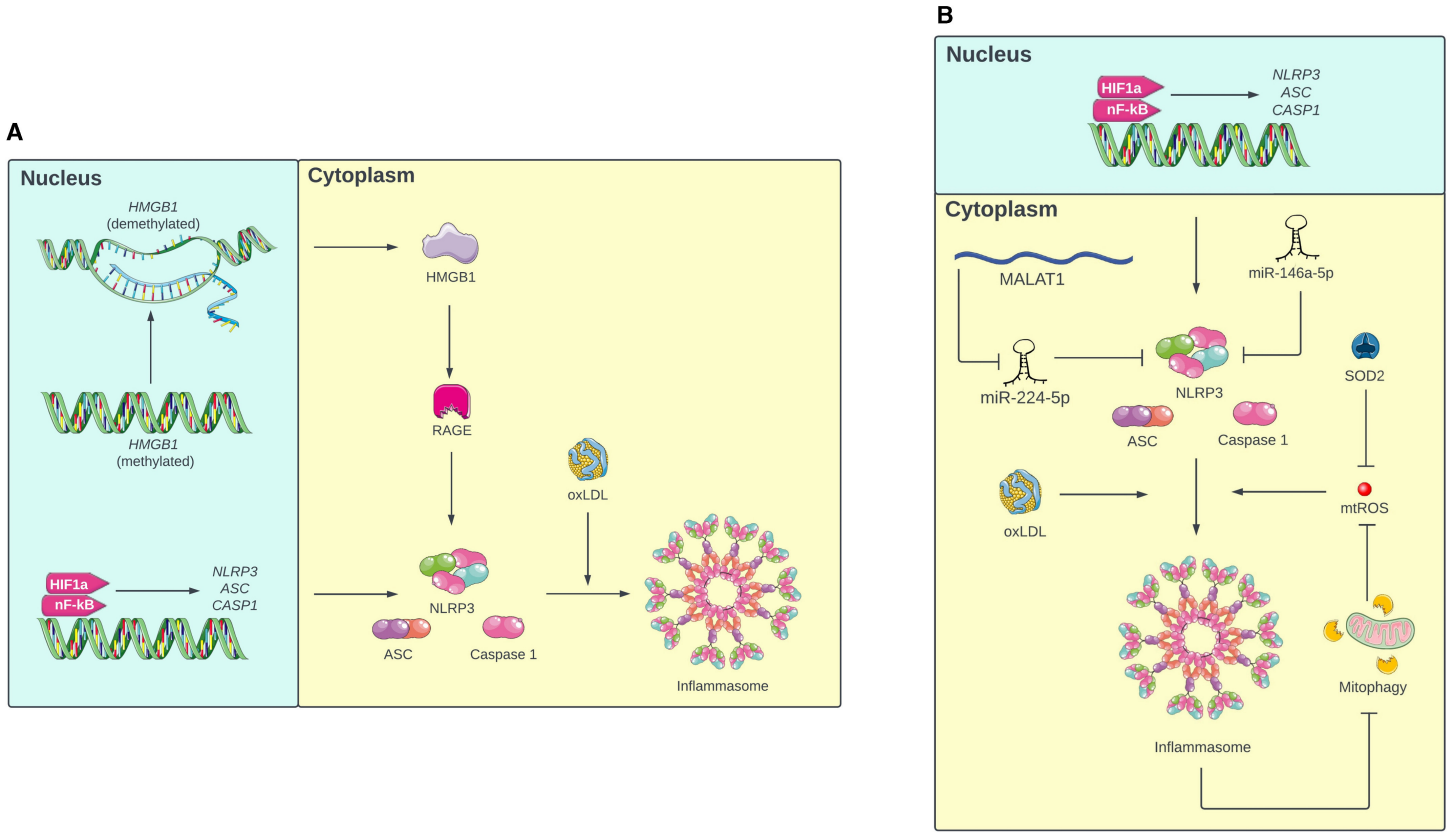
**(A)** Mechanism of inflammasome activation in monocytes under intermittent hypoxia (IH). First, IH primes the inflammasome by increasing transcription of NLRP3, ASC, and caspase 1 via HIF-1a and nF-KB. In response to oxLDL, these components oligomerize to form the active inflammasome. Additionally, IH inhibits methylation of the *HMGB1* gene, resulting in expression of HMGB1, which increases expression of inflammasome components via RAGE. **(B)** Mechanism of inflammasome activation in macrophages under IH. In addition to the pathway involving HIF-1a, nF-KB, and oxLDL, inflammasome oligomerization in macrophages is also mediated by mitochondrial ROS (mtROS). The inflammasome promotes its own activation by inhibiting mitophagy, which further increases mtROS levels. Additionally, IH increases NLRP3 expression by inhibiting miR-224-5p, a miRNA that targets NLRP3, via MALAT1. IH also decreases levels of miR-146a-5p and SOD2, which prevent inflammasome activation by targeting NLRP3 and mtROS respectively. Portions of the figure utilized images from Servier Medical Art, licensed under Creative Commons Attribution 4.0 International.

There is strong evidence that OSA contributes to inflammasome activation in monocytes and macrophages (Figure 1). Peripheral blood monocytes from individuals with OSA had elevated expression of NLRP3, ASC, and caspase 1, as well as higher levels of IL-18 and IL-1β in serum (Diaz-Garcia et al., 2022, 2023; Zong et al., 2023). Several studies also found that microglia undergo inflammasome activation when cultured under IH (Gong et al., 2020; Wu et al., 2021; Du et al., 2020). Interestingly, individuals with OSA did not have elevated serum levels of NLRP3, ASC, and caspase-1, despite higher serum IL-18 and IL-1β levels (Tang et al., 2019). This suggests that inflammasome activation is an intracellular process that results in the release of IL-18 and IL-1β.

Inflammasome activation in monocytes usually involves two steps: priming and full activation. During priming, the expression of NLRP3, ASC, and caspase 1 increases, but NLRP3 remains inactive, preventing oligomerization. When additional cellular damage is signaled, NLRP3 enters the active state, which allows NLRP3, ASC, and caspase 1 to oligomerize and form the inflammasome. The inflammasome then cleaves immature IL-18 and IL-1β into their mature forms and triggers their release from the cell (Swanson et al., 2019). Diaz-Garcia et al. (2022) proposed a model for how this two-step activation process occurs in OSA: first, IH primes the inflammasome by activating transcription factors hypoxemia inducible factor-1a (HIF-1a) and nuclear factor-kappa B (NF-kB), which increase expression of NLRP3, ASC, and caspase 1. Concurrently, IH causes cellular damage, which increases levels of oxidized low-density lipoprotein (oxLDL) in circulation. When NLRP3 senses the circulating oxLDL, the inflammasome is fully activated. In support of this model, experimental results demonstrated that both IH and oxLDL were required to induce IL-1β release in human monocytes (Diaz-Garcia et al., 2023). Additionally, the HMGB1/RAGE/NLRP3 pathway is another proposed mechanism of inflammasome activation in monocytes. Tan et al. (2023) found that chronic IH inhibits methylation of the high mobility group box 1 (HMGB1) gene in monocytes, which increases the expression of HMGB1 protein. HMGB1 then increases the expression of the receptor for advanced glycation endproducts (RAGE), which promotes expression of NLRP3, ASC, and caspase 1.

In macrophages, inflammasome activation involves a two-step mechanism similar to inflammasome activation in monocytes (Diaz-Garcia et al., 2022). Fitzpatrick et al. (2021) found that in contrast to lean mice, bone marrow derived macrophages (BMDMs) from mice with obesity secreted IL-1β when cultured under IH. This suggests that IH alone is insufficient for full inflammasome activation. The mice with obesity likely had elevated levels of oxLDL and other cellular damage signals, which were necessary to induce inflammasome oligomerization and cytokine release under IH conditions.

Inflammasome activation in macrophages also involves mitochondrial damage and oxidative stress. IH causes mitochondrial damage, resulting in the production of mitochondrial reactive oxygen species (mtROS) and oxidized mitochondrial DNA (mtDNA), both of which activate the inflammasome (Zhou et al., 2011). Therefore, mitophagy (the selective autophagy of damaged mitochondria) can clear mtROS and oxidized mtDNA from the cell and thus decrease inflammasome activation (Gong et al., 2020). In microglia, Gong et al. (2020) found that pinocembrin (a flavonoid antioxidant) can upregulate mitophagy, resulting in lower mtROS levels and less inflammasome activation. Interestingly, inflammasome inhibition in microglia resulted in increased mitophagy (Wu et al., 2021), which suggests a bidirectional relationship between mitophagy and the inflammasome. Once activated by mtROS and oxidized mtDNA, the inflammasome inhibits mitophagy, which results in further accumulation of mtROS and oxidized mtDNA, leading to even more inflammasome activation.

In addition to mitophagy, manganese superoxide dismutase (SOD2) is a mitochondrial antioxidant enzyme that provides another source of protection against mtROS (Flynn and Melov, 2013). Fu et al. (2020) and Song et al. (2020) found that mice exposed to IH had low levels of SOD2, leading to higher oxidative stress and inflammasome activation in interstitial macrophages. This suggests that IH activates the inflammasome by not only increasing oxidative stress, but also downregulating antioxidative processes. The RNA-RNA interactome may also mediate OSA-induced inflammasome activation in macrophages. In microglia, Du et al. (2020) found that IH increases the expression of MALAT1, a lncRNA that inhibits miR-224-5p, a miRNA that represses NLRP3 translation. Similarly, Zhang et al. (2023) found that the level of miR-146a-5p, another miRNA that targets NLRP3, in microglial exosomes decreased in mice exposed to chronic IH. In both cases, failure of miRNA to inhibit NLRP3 contributed to inflammasome activation in microglia, resulting in neuroinflammation.

Overall, IH is a major contributor to inflammasome activation in immune cells. However, there is a gap in the literature that compares inflammasome activation pathways in monocytes and macrophages. For example, several studies characterize the role of mitophagy and oxidative stress in macrophages, but not monocytes. Furthermore, it is unclear whether inflammasome activation occurs through a similar mechanism in macrophages that reside in different tissues. Therefore, comparative studies involving multiple immune cell types, including tissue-specific macrophages and monocytes, would be invaluable for uncovering the distinct or shared mechanisms of inflammasome activation.

## 3 The inflammasome and OSA complications

### 3.1 Neurocognitive complications

**Figure 2:**
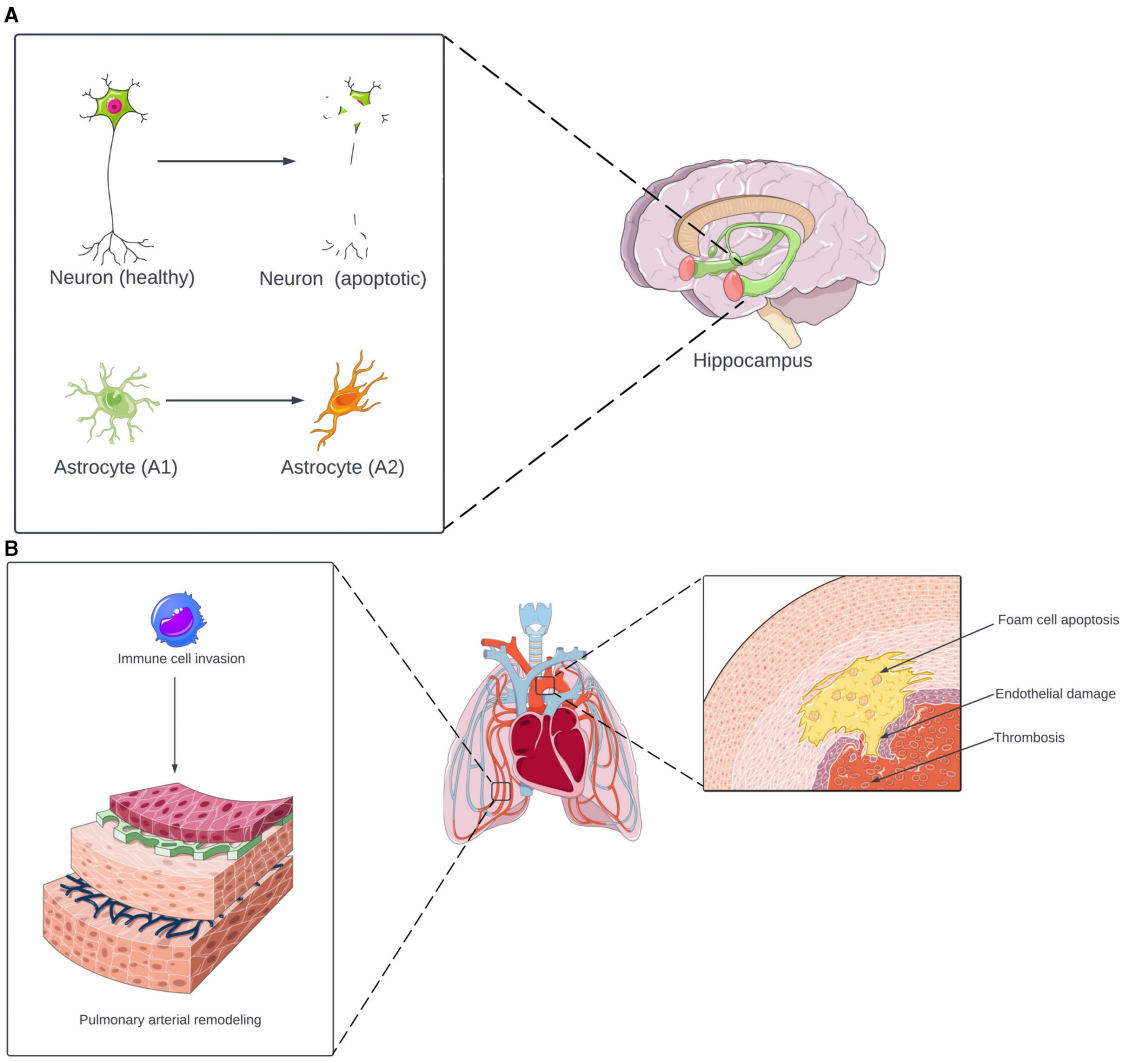
**(A)** Neurological effects of inflammasome activation in OSA. Under OSA-induced inflammasome activation, neurons in the hippocampus undergo apoptosis. The inflammasome also induces astrocyte transformation from the neuroprotective A1 phenotype to the neurotoxic A2 phenotype. The combination of neuronal apoptosis and astrocyte-induced inflammation contributes to the development of cognitive and memory deficits. **(B)** Cardiovascular effects of inflammasome activation in OSA. The inflammasome promotes a triad of thrombosis, endothelial damage, and foam cell apoptosis, increasing the risk of atherosclerosis and other cardiovascular diseases. In the lungs, inflammasome activation induces monocytes and macrophages to invade the pulmonary vasculature. This results in pulmonary arterial remodeling, which increases the risk of pulmonary hypertension. Portions of the figure utilized images from Servier Medical Art, licensed under Creative Commons Attribution 4.0 International.

There is strong evidence for the association between OSA and neurocognitive complications, including deficits in memory and focus (Gildeh et al., 2016). Animal models suggest that the inflammasome may contribute to developing these complications (Figure 2). In rodents exposed to IH, inflammasome activation was associated with neuronal apoptosis in the hippocampus, as well as poor performance on the contextual fear conditioning, spatial learning, and memory tests (Gong et al., 2020; Wu et al., 2021; Zhang et al., 2023) and with a transformation from A2 (neuroprotective) astrocytes to A1 (neurotoxic) astrocytes, resulting in damage to the hippocampus and impaired spatial learning (She et al., 2022). Overall, these animal studies suggest that the inflammasome may also contribute to the development of cognition and memory issues in individuals with OSA.

### 3.2 Cardiovascular complications

The development of cardiovascular complications in individuals with OSA may also be associated with inflammasome activation (Figure 2) (Kohler and Stradling, 2010; Gaisl et al., 2015). In a canine model of OSA, Yu et al. (2017) found that inflammasome activation is associated with aortic damage. Additionally, the inflammasome induces peripheral blood monocytes to transform into foam cells and undergo apoptosis, resulting in plaque depositions within arteries (Tan et al., 2023). Furthermore, inflammasome activation induces high levels of tissue factor (TF) release from macrophages, which promotes coagulation and thrombosis (Diaz-Garcia et al., 2022, 2023). There is also a link between the inflammasome and pulmonary hypertension (PH), a known complication of OSA. In mice exposed to IH, inflammasome activation induced monocyte and macrophage invasion of the lungs, which triggered pulmonary vascular remodeling, ultimately leading to PH (Song et al., 2020; Fu et al., 2020). Overall, the inflammasome is a potential link between OSA and cardiovascular complications, promoting vascular injury, atherosclerosis, hyper-coagulability, and PH.

## 4 The inflammasome and OSA therapeutics

### 4.1 CPAP

The current first-line treatment for OSA is continuous positive airway pressure (CPAP). CPAP improves daytime function and decreases the risk of cardiovascular complications in individuals with OSA (Patel et al., 2003; Haentjens et al., 2007). However, compliance is a major limitation of CPAP, as < 50% of individuals are adherent to therapy, with decreased adherence in individuals with lower income (Pandey et al., 2020). Barriers to adherence include limited medical insurance coverage for the device, as well as lack of access to add-on accessories like mask liners and head gear that improve usability (Pandey et al., 2020). Low adherence to CPAP therapy is concerning because untreated or partially treated OSA can exacerbate the risk of cardiovascular events. Moreover, for individuals with overweight or obesity, CPAP alone may not be sufficient to prevent cardiovascular complications; concurrent weight loss or antihypertensive medication are also required (Levy et al., 2015). These limitations highlight the urgent need for alternative or adjunct therapies that are more acceptable to patients and target the underlying mechanisms of OSA-related inflammation and cardiovascular risk.

Despite these limitations, there is evidence that CPAP reduces airway inflammation. Among individuals with OSA, CPAP use was associated with lower levels of inflammatory proteins in the upper airway (Cohen et al., 2023) and lower levels of TNF-α in exhaled breath condensate (Lin et al., 2016). However, the relationship between CPAP and systemic inflammation is unclear. While CPAP use was associated with lower levels of inflammatory markers like IL-18, TNF-α, and C-reactive protein (CRP) in blood (Steiropoulos et al., 2009; Jin et al., 2017), suggesting that CPAP decreases systemic inflammation, one meta-analysis found that CPAP only reduced blood CRP levels when combined with weight loss (Chirinos et al., 2014). Moreover, others reported no association between CPAP use and inflammatory markers in blood (Colish et al., 2012; Thunstrom et al., 2019; Karamanli et al., 2014; Vgontzas et al., 2008; Wali et al., 2021; Kritikou et al., 2014; Kohler et al., 2009).

The impact of CPAP on the inflammasome has been less studied. Notably, Hall et al. (2024) found that CPAP use was associated with lower plasma levels of hsa-miR233-3p and hsa-miR233-5p, two miRNAs that promote inflammasome activation. These findings suggest a potential role for CPAP in modulating inflammasome activity, but further research is needed to fully understand the mechanisms involved and to clarify the broader implications for systemic inflammation.

### 4.2 GLP-1 receptor agonists

Glucagon-like peptide-1 receptor agonists (GLP-1RAs), including tirzepatide, liraglutide, and exanetide, are effective treatment options for type-2 diabetes (T2D) and obesity. They decrease blood glucose levels and promote weight loss by inducing insulin secretion and delaying gastric emptying (Mariam and Niazi, 2024). GLP-1RAs have demonstrated significant therapeutic potential for individuals with OSA. The SURMOUNT-OSA and SCALE clinical trials found that GLP-RAs (tirzepatide and liraglutide, respectively) significantly lowered OSA severity and body weight in adults with comorbid OSA and obesity (Malhotra et al., 2024; Blackman et al., 2016). One proof of concept study of liraglutide vs. CPAP vs. the combination of liraglutide and CPAP found that CPAP alone was associated with greater improvement in vascular inflammation and early atherosclerosis compared to liraglutide alone and combination treatment (O'Donnell et al., 2024). However there were significant limitations to this study including a sample size of only 30 among 3 treatment arms. CPAP adherence was an hour lower in the combination group compared to CPAP alone which may explain why the CPAP alone arm was significantly better compared to combination therapy. This finding does highlight that while GLP-1RAs are highly promising, PAP therapy in individuals with high adherence is likely to remain as the most efficacious therapy for OSA. However, for individuals unable to tolerate PAP, GLP-1RAs are emerging as an important additional treatment strategy. The therapeutic efficacy of GLP-1RAs for individuals with OSA is being further studied in ongoing clinical trials. The ROMANCE study is evaluating how individuals with concurrent OSA, obesity, and T2D respond to liraglutide alone or in combination with CPAP (Sprung et al., 2020). The STRIVE study is an open-label, real-world randomized control trial assessing the financial burden and practical implications of daily liraglutide administration to patients with obesity, prediabetes, T2D, hypertension, and/or OSA (Papamargaritis et al., 2020).

The mechanism through which GLP-1RAs alleviate OSA severity remains unclear. Related neuropeptides such as leptin and orexin have been shown to play a role in breathing and upper airway muscle tone (Shapiro et al., 2014; Gestreau et al., 2008). However, there is insufficient data to determine if GLP-1RAs directly affect control of breathing or upper airway patency. Currently, given the well-established role of excess body weight in OSA pathogenesis, the weight-loss effects of GLP-1RAs are widely regarded as the primary driver of their benefits for OSA (Le et al., 2024). This is supported by findings from the SCALE trial, which demonstrated a proportional relationship between the extent of weight loss and reductions in OSA severity (Blackman et al., 2016). Similarly, improvements in OSA symptoms have been observed with other weight-loss interventions, such as bariatric surgery and lifestyle modifications (Foster et al., 2009; Dixon et al., 2012). However, GLP-1RAs exhibit pleiotropic effects, including anti-inflammatory properties, suggesting that their benefits may extend beyond weight loss (Papaetis, 2023). By mitigating oxidative stress from intermittent hypoxia and reducing inflammation, GLP-1RAs may help alleviate OSA-associated complications, particularly cardiovascular risks. For instance, the SURMOUNT-OSA trial found that tirzepatide significantly reduced plasma levels of C-reactive protein, a marker associated with systemic low-grade inflammation and cardiovascular disease (Malhotra et al., 2024). Interestingly, GLP-1RAs may also inhibit the inflammasome. In a meta-analysis, Bray et al. (2021) found that GLP-1RAs decreased plasma levels of malondialdehyde, an oxidative stress marker. Therefore, GLP-1RAs could reduce the oxidative stress generated by IH, potentially decreasing inflammasome activation in individuals with OSA. Additionally, Tremblay et al. (2014) and Zobel et al. (2021) found that GLP-1RAs lowered the plasma levels of IL-18 and IL-1β in individuals with T2D, which suggests that GLP-1RAs could prevent the inflammasome from cleaving and releasing pro-inflammatory cytokines into the bloodstream.

Animal studies provide further evidence that GLP−1RAs inhibit the inflammasome. Liraglutide decreased expression of NLRP3, IL-18, and IL-1β in mice and pigs with diabetes (Chen et al., 2021; Xia et al., 2020). Additionally, liraglutide and exenatide decreased hepatic steatosis by inhibiting the inflammasome in mice with non-alcoholic fatty liver disease (Zhu et al., 2018; Yu et al., 2019; Shao et al., 2018). In microglial cells, Jia et al. (2024) found that liraglutide inhibited the inflammasome by promoting mitophagy, which reduced oxidative stress. GLP-1RAs can also downregulate key activators of the inflammasome, including HIF-1a, NF-kB, and TXNIP (Marlet et al., 2018; Zhou et al., 2020). Thus, beyond their primary role in promoting weight loss, GLP-1RAs may offer additional benefits through inflammasome inhibition, potentially mitigating the inflammatory complications associated with OSA.

## 5 Conclusion

Through its hallmark features of IH and reoxygenation, OSA drives inflammasome activation in immune cells, including monocytes, macrophages, and microglia. This activation occurs via multiple mechanisms: IH induces a two-step process that first increases the expression of inflammasome components, which subsequently oligomerize in response to circulating lipids and lipoproteins. Additionally, IH contributes to further inflammasome activation through mitochondrial damage, oxidative stress, and RNA-RNA interactions. As a result, activated immune cells cause systemic inflammation, which is a key contributor to the neurocognitive and cardiovascular complications frequently observed in individuals with OSA.

Despite the widespread recognition of CPAP as a cornerstone of OSA treatment, its impact on systemic inflammation and inflammasome activity remains poorly understood. Preliminary findings, specifically the reduction of inflammasome-associated miRNAs with CPAP use, indicate a potential anti-inflammatory effect that warrants further investigation. Additionally, GLP-1RAs, known for their role in weight management and T2D, may also inhibit the inflammasome. Therefore, these drugs can help prevent the neurological and cardiovascular complications of OSA that arise from inflammasome activation.

However, significant gaps in our understanding persist. Specifically, the relationship between CPAP and systemic inflammation and the mechanisms by which CPAP influences inflammasome activity require further study. Furthermore, most research on GLP-1RAs and the inflammasome has been conducted in the context of T2D, leaving their effects in individuals with OSA less explored. Addressing these gaps is crucial for advancing our understanding of OSA pathophysiology and for developing targeted therapies that could improve patient outcomes.

In summary, the interplay between OSA and the inflammasome is linked to the development of numerous systemic complications including cardiovascular disease and neurocognitive impairment, which exacerbate the overall burden of the disorder. Table 1 provides a summary of the existing literature on the relationship between OSA and the inflammasome. A deeper understanding of inflammasome activation in OSA and its modulation by existing and emerging therapies could lead to more effective management strategies, ultimately improving the quality of life for individuals with OSA.

**Table 1:** Overview of major findings regarding inflammasome activation in monocytes and macrophages, neurocognitive, and cardiovascular complications of inflammasome activation in OSA, and the interaction between CPAP and GLP-1RAs and the inflammasome.

**Topic**	**Study type**	**Finding**
Inflammasome activation in monocytes	Human	Peripheral blood monocytes from patients with OSA had elevated levels of inflammasome components and inflammatory cytokines (Diaz-Garcia et al., 2022, 2023; Zong et al., 2023).
	Cell culture	Intermittent hypoxia (IH) and oxidized low-density lipoprotein (oxLDL) were both required for inflammasome activation in cultured monocytes (Diaz-Garcia et al., 2023).
	Animal and cell culture	IH induced inflammasome activation through HMGB1 and RAGE in cultured monocytes and mice (Tan et al., 2023).
Inflammasome activation in macrophages	Cell culture	IH induced inflammasome activation in cultured microglia (Gong et al., 2020; Wu et al., 2021; Du et al., 2020).
	Cell culture	Macrophages from mice with obesity, but not lean mice, underwent inflammasome activation when cultured under IH (Fitzpatrick et al., 2021).
	Cell culture	Pinocembrin reduces inflammasome activation by promoting mitophagy in cultured microglia (Gong et al., 2020).
	Cell culture	Inflammasome activation inhibits mitophagy, resulting in mitochondrial ROS accumulation in cultured microglia (Wu et al., 2021).
	Animal	In mice, IH exposure is associated with downregulation of the antioxidant enzyme SOD2, promoting inflammasome activation in interstitial macrophages (Song et al., 2020; Fu et al., 2020).
	Animal	In microglia of mice exposed to IH, there were lower levels of miR-224-5p and miR-146a-5p, which are miRNAs that target NLRP3 (Zhang et al., 2023; Du et al., 2020).
Neurocognitive complications of inflammasome activation	Animal	Inflammasome activation in mice exposed to IH is associated with hippocampal damage and memory deficits (Gong et al., 2020; Wu et al., 2021; Zhang et al., 2023).
	Animal	Inflammasome activation in rats exposed to IH is associated with a shift from neuroprotective (A2) to neurotoxic (A1) astrocytes (She et al., 2022).
Cardiovascular complications of inflammasome activation	Human (review)	Oxidative stress and systemic inflammation are associated with vascular damage in OSA patients (Kohler and Stradling, 2010; Gaisl et al., 2015).
	Animal	Inflammasome activation is associated with aortic damage in a canine model of OSA (Yu et al., 2017).
	Animal	In mice exposed to IH, inflammasome activation promotes foam cell apoptosis and plaque deposition in arteries (Tan et al., 2023).
	Human	In patients with OSA, inflammasome activation induces monocytes to release tissue factor (TF) into the bloodstream, which increases risk of thrombosis (Diaz-Garcia et al., 2022, 2023).
	Animal	In a mouse model of OSA, inflammasome activation promotes immune cell invasion of the lungs, leading to pulmonary hypertension (Song et al., 2020; Fu et al., 2020).
The inflammasome and CPAP	Human	In patients with OSA, CPAP use is associated with decreased airway inflammation (Cohen et al., 2023; Lin et al., 2016).
	Human	In patients with OSA, CPAP use is associated with decreased levels of inflammatory markers IL-18, TNF-α, and C-reactive protein (CRP) in blood (Steiropoulos et al., 2009; Jin et al., 2017).
	Human	In patients with OSA, CPAP use was not effective at reducing systemic inflammation unless it was combined with weight loss (Chirinos et al., 2014).
	Human	In patients with OSA, there is no association between CPAP use and systemic inflammation (Colish et al., 2012; Thunstrom et al., 2019; Karamanli et al., 2014; Vgontzas et al., 2008; Wali et al., 2021; Kritikou et al., 2014; Kohler et al., 2009).
	Human	In patients with OSA, CPAP use was associated with lower plasma levels of hsa-miR233-3p and hsa-miR233-5p, two miRNAs that promote inflammasome activation (Hall et al., 2024).
The inflammasome and GLP-1 receptor agonists	Human	Tirzepatide and liraglutide lowered body weight, OSA severity, and serum C-reactive protein levels in adults with OSA and obesity (Malhotra et al., 2024; Blackman et al., 2016).
	Human	Compared with liraglutide and CPAP and liraglutide alone, CPAP alone was associated with a greater reduction in vascular inflammation and early atherosclerosis (O'Donnell et al., 2024).
	Human (meta-analysis)	GLP-1RA use is associated with decreased plasma levels of malondialdehyde, suggesting that GLP-1RAs reduce oxidative stress (Bray et al., 2021).
	Human	In patients with type 2 diabetes, GLP-1RAs lowered the plasma levels of IL-18 and IL-1β (Tremblay et al., 2014; Zobel et al., 2021).
	Animal	Liraglutide decreased expression of NLRP3, IL-18, and IL-1β pigs and mice with diabetes (Chen et al., 2021; Xia et al., 2020).
	Animal	Liraglutide promotes mitophagy in mouse microglia (Jia et al., 2024).
	Animal (meta-analysis)	A meta-analysis of post-stroke liraglutide administration in rodents shows that liraglutide downregulates HIF-1a and NK-kB, which are key activators of the inflammasome (Marlet et al., 2018).
	Animal	Liraglutide administration in mice after acute lung injury results in downregulation of TXNIP, a key activator of the inflammasome (Zhou et al., 2020).

The findings are grouped by topic. The second column indicates whether the study used was conducted on cell cultures, animal models, or human subjects. The third column gives a brief summary of the study findings.
